# Ridge Minimization of Ablated Morphologies on ITO Thin Films Using Squared Quasi-Flat Top Beam

**DOI:** 10.3390/ma11040530

**Published:** 2018-03-30

**Authors:** Hoon-Young Kim, Jin-Woo Jeon, Wonsuk Choi, Young-Gwan Shin, Suk-Young Ji, Sung-Hak Cho

**Affiliations:** 1 Department of Laser & Electron Beam Application, Korea Institute of Machinery & Material (KIMM), 171 Jang-dong, Yuseong-gu, Daejeon 305-343, Korea; hykim@kimm.re.kr (H.-Y.K.); jwj@kimm.re.kr (J.-W.J.); cws@kimm.re.kr (W.C.); sinyg90@kimm.re.kr (Y.-G.S.); ji10047@kimm.re.kr (S.-Y.J.); 2 Department of Nano-Mechatronics, Korea University of Science & Technology (UST), 176 Gajung-dong, Yuseong-gu, Daejeon 305-343, Korea

**Keywords:** femtosecond laser, ITO thin film, slit, ridge minimization, ablation

## Abstract

In this study, we explore the improvements in pattern quality that was obtained with a femtosecond laser with quasi-flat top beam profiles at the ablated edge of indium tin oxide (ITO) thin films for the patterning of optoelectronic devices. To ablate the ITO thin films, a femtosecond laser is used that has a wavelength and pulse duration of 1030 nm and 190 fs, respectively. The squared quasi-flat top beam is obtained from a circular Gaussian beam using slits with varying *x*-*y* axes. Then, the patterned ITO thin films are measured using both scanning electron and atomic force microscopes. In the case of the Gaussian beam, the ridge height and width are approximately 39 nm and 1.1 μm, respectively, whereas, when the quasi-flat top beam is used, the ridge height and width are approximately 7 nm and 0.25 μm, respectively.

## 1. Introduction

Indium tin oxide (ITO) thin films are the primary materials for the production of transparent electrodes used in organic light-emitting diodes (OLEDs), solar cells, liquid crystal displays (LCDs), and other optical devices, because of their good electrical conductivity, transparency in the visible spectrum, high infrared reflectivity, low resistivity, high carrier concentrations, high mobility rates, good adhesion ability on glass, and excellent chemical stability. In order to reduce the resistive losses and lost active areas in optoelectronic devices, high quality-patterning of these ITO thin films is required for the formation of interconnect lines and display assemblies [1,2,3]. In addition, owing to its high optical transmittance and conductivity, patterned ITO has been applied as an electrode material to enhance the uniformity of the current spread. Recently, manufacturers of smartphones have tried to reduce the thickness of ITO layers in smartphone touch screens to decrease not only the touch screen thickness, but also the weight, and consequently, the price of smartphones [4]. However, with a reduction in touch screen thickness by reducing the thickness of ITO layers, certain technical issues emerge that need to be solved; for example, a reduction in luminous efficiency, increased power consumption, and reduction of usage lifetime [5]. When considering this, ridge minimization of patterned ITO is an optimization method that can increase the luminous efficiency of these devices. Thus, well-defined edges and good electrical isolation with a narrow separation between the conductor lines are essential requirements, particularly when high precision and positional accuracy in the order of a few micrometers is necessary in touch panel and OLED manufacturing.

The patterning and structuring of the ITO electrodes was conventionally performed by a photolithography process that is based on etching in acidic solutions [6]. However, this conventional photolithographic method can lead to increased manufacturing costs, because it involves multiple steps and the use of hazardous acid, which can cause environmental problems, and thus, incur significant disposal costs [7,8]. In addition, a uniform pattern formation is hampered by the process of wet etching, which critically depends on the characteristics of the materials that are used, such as their crystallinity and microstructure, imposing stringent limitations on the production process of the ITO films [9]. Thus, to reduce the manufacturing costs that are associated with ITO film production, which will promote its share in the display market, it is necessary to develop a non-lithographic or direct patterning strategy to fabricate fine structures with well-defined edges on the ITO electrodes. Various techniques have been developed in the past to manufacture ITO electrodes with well-defined edges and electrically insulated grooves between the conductor lines for optoelectronic devices; in particular, the laser direct patterning technique is an etching method that can not only be effectively used instead of the conventional wet photolithography method, but can also lower the cost of manufacturing [7,10].

These laser direct patterning technologies, which employ the rapid scanning of micro-chemical etching reactions, are important for mask-less non-lithographic fabrication of microelectronics, especially optoelectronic devices [11]. The laser direct patterning method is a dry etching method, and it has been used in many studies to produce a circuit pattern on transparent conductive oxide films [12,13]. The advantages of laser processing include non-contact processing, absence of tool wear, small-scale chipping, considerably small thermal effect, production flexibility, high speed, high accuracy, and low environmental pollution, among others [14]. Laser ablation is the removal of materials from a substrate by the direct absorption of laser energy, thus producing the desired combination of narrow and clean patterning, because of the localized heating and material removal; therefore, laser processing technology is naturally used in patterning ITO film by selectively removing the ITO material and retaining the desired pattern on the ITO film [15]. In addition, pulse lasers with varying pulse durations can be used to pattern ITO thin films on a glass substrate. Aside from the abovementioned cases, well-defined patterns could be created with an increasing pattern quality by decreasing the pulse width of the laser that is used; however, on using the laser patterning method, high height spikes remain at the edges of the ablated pattern because of the considerable amount of ITO melt that is generated during laser irradiation, which is arguably the most serious disadvantage of this method [12]. Therefore, the removal of material is calculated in atomic or molecular units. Plasma, composed of molecules, atoms, and electrons removed by high energy density in the ablation area, is formed. After the plasma disappears, unstable atoms or molecules combine to become nano-sized particles. Nanoparticles attach to the vicinity of the ablation area. In the case of a pattern with a narrow line width, these particles may cause the circuit to be open or shorted. Further, in case of the pattern for laminating thin films, the upper layers may not be formed correctly by the nanoparticle height, and shorting may occur between the lower floor and upper floor [16,17,18]. Moreover, selective laser patterning of ITO with excellent electronic isolation properties and minimal damage to the glass substrates is required for high volume production; in that light, the laser patterning processes that operate with energy densities close to the threshold fluence, allow for the optimal utilization of the laser energy and ensure minimal damage to the glass substrate [10,19,20].

Nevertheless, significant ridges were formed at the edges of the laser ablated ITO lines; the formation of these ridges could be attributed to the lack of thermal vaporization at the edge of the molten laser-irradiated film [21,22]. Owing to this, elevated ridges on the edges as well as the ITO residue at the bottom of an ablated surface can easily occur, which may cause the shorting of the structure and the adjacent electrodes; consequently, leading to a reduction in the longevity and efficiency of these devices [23]. In particular, factors, such as poor edge quality and substrate damage, increase the visibility of the patterned feature as well as the device failure rates [24]. To overcome the abovementioned disadvantages, Krause et al. used a femtosecond laser to remove the ITO, which was irradiated through the glass side to create almost-rectangular cross-sectional groove profiles, unlike in the case of a Gaussian laser beam [25]. In addition, other researchers utilized a nanosecond laser to pattern α-ITO thin films using the crystallization effect [26]; however, this method also has a number of limitations, especially for devices that are fabricated directly onto the substrate, because it can easily lead to material accumulation at the edge of the ablation trench. The modern OLED and radio-frequency infrared (RFID) devices boast strict specifications for the shape and accuracy of ITO processing, especially, the requirement of well-defined, sharp edges; these sharp edges for the ablated lines are considerably important in the case of organic electronics devices, because these are thin film structures with a total thickness of the active films in the range of 100 nm [27]. Consequently, minimizing the crater ridge height decreases line visibility as well as the device failure rates.

In general, ultrashort pulsed laser ablation leads to small thermally induced defects in the remaining material; the thermal defects are often difficult to avoid with longer laser pulses. In laser material processing, ultrashort pulses create little or no heat-affected zones around the processed area; therefore, there is no cracking, and less debris is generated when compared with irradiation by long pulses [28]. Thus, femtosecond and picosecond lasers have been utilized to scribe ITO films [29]. Recent investigations on picosecond laser scribing of ITO films demonstrated that material damage threshold depends on the wavelength and repetition rate of lasers as well as other system parameters [30]. When compared with the picosecond laser pulses, femtosecond laser can induce non-thermal structural changes owing to electronic excitation. Therefore, the femtosecond laser has been used for precision material removal in the field of fabrication research for optoelectronic devices, such as OLEDs and solar cells.

This study presents a method to minimize ablated ridges in ITO thin film using quasi-flat top beams by controlling the number of femtosecond pulses. The quasi-flat top beam is generated from a Gaussian beam by passing it through a slit. Furthermore, the morphologies of the ablated edges and ridges were characterized with both, an atomic force microscope (AFM) and a scanning electron microscope (SEM).

## 2. Experimental Setup

**Figure 1:**
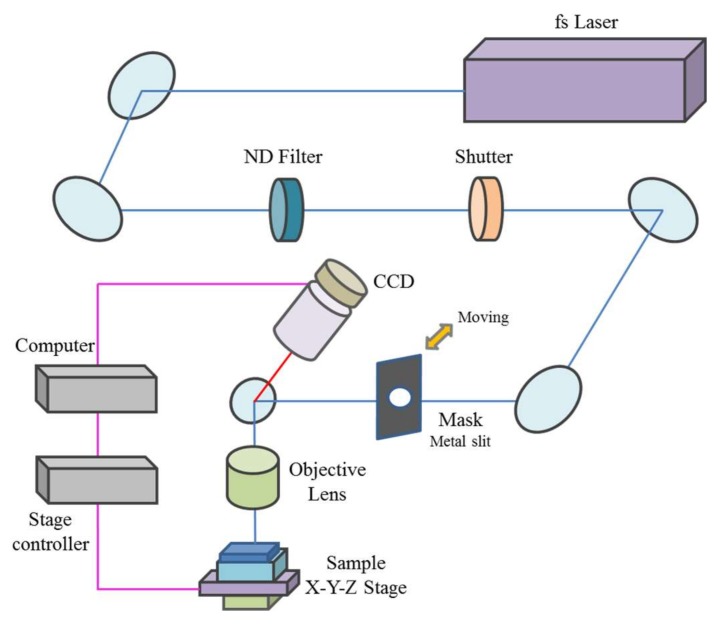
Schematic of the femtosecond laser patterning system.

In our experiments, ITO thin films with a nominal thickness of 140 nm and a transmittance of 80% (In_2_O_3_:SnO_2_ = 90:10) deposited onto glass substrates using a DC magnetron sputtering system were used. Then, the six sides of the glass substrate were optically polished. A sheet resistance of 8 Ω/sq. was used in the experiments. The surface characteristics of the ablated ITO thin films were observed using an SEM (S-4800, Hitachi, Tokyo, Japan), and the three-dimensional morphologies and cross-sectional graph of the ablated ITO thin film patterns were prepared using the AFM (NX-10, Park Systems, Suwon, Korea). A schematic diagram of the experimental setup is shown in Figure 1. In our experiments, a commercial regenerative amplified mode-locked Yb:KGW laser (S-Pulse HR, Amplitude Systèmes, Pessac, France) with a 1030 nm central wavelength, pulse duration of 190 fs, repetition rate of 30 kHz, and a maximum pulse energy of 66 μJ, was used to pattern the ITO thin films. The laser power was controlled using a neutral-density filter, and the pulse energy was calculated after measuring the laser power using a power meter. Depending on the experimental setup, either a Gaussian beam profile or a quasi-flat top beam profile was used. The beam M^2^ quality parameter was 1.2, and the laser beam was focused using an objective lens with a numerical aperture of 0.42 (M Plan Apo NIR 20X, Mitutoyo, Kanagawa, Japan). Furthermore, in the case of the laser beam with a Gaussian beam profile, it was delivered onto the sample through the optical system. The ITO thin film sample was fixed on a micro-positioning stage controlled by a computer, and the stage could be moved in along the *x*-, *y*-, and *z*-axes. The femtosecond laser beam was linearly polarized and had a spatially Gaussian beam profile. As shown in Figure 1, the Gaussian beam was shaped via the slit to have a quasi-flat top; during which the circle Gaussian beam was changed to a square quasi-flat top beam because the beam was cut with a slit.

## 3. Results and Discussion

Through the experiments, it was observed that the targeted material was only removed if the fluence reached the ablation threshold, which is strongly dependent on the characteristics of the target material. In the case of a multilayer material, the ablation threshold for each layer is important for the selective ablation of the desired layer; when considering that OLEDs and solar cells are composed of multilayer structures, patterning without damage to the underlying layer is important in the industry. Thus, the ablation threshold of the ITO thin film and the glass substrate were measured to conduct selective removal successfully; in particular, the ablation threshold of the ITO thin films was measured as 0.24 J/cm^2^ at 190 fs; in addition, the measured ablation threshold of the glass substrate was 2.1 J/cm^2^. Bian et al. reported that the ablation threshold of ITO thin films was 0.3 J/cm^2^, with a pulse duration of 60 fs [31], whereas Cheng et al. reported that the ablation threshold of ITO thin films was 0.31 J/cm^2^ at 120 fs [32]. Furthermore, McDonnell et al. reported that the ablation threshold of ITO thin films was 0.26 J/cm^2^, with a pulse duration of 500 fs [24]. These differences in ablation thresholds could be explained by the different etching rates because of the distinct nature of each ITO thin film or the differences in the exact pulse shape of the femtosecond laser used in these ablation experiments. In this study, the patterning was conducted using single pulse femtosecond irradiation with Gaussian and quasi-flat top beam profiles being passed through a slit at 0.9 J/cm^2^ in order to avoid damage to the glass surface while achieving well-defined ablation edges on the ITO thin film.

**Figure 2:**
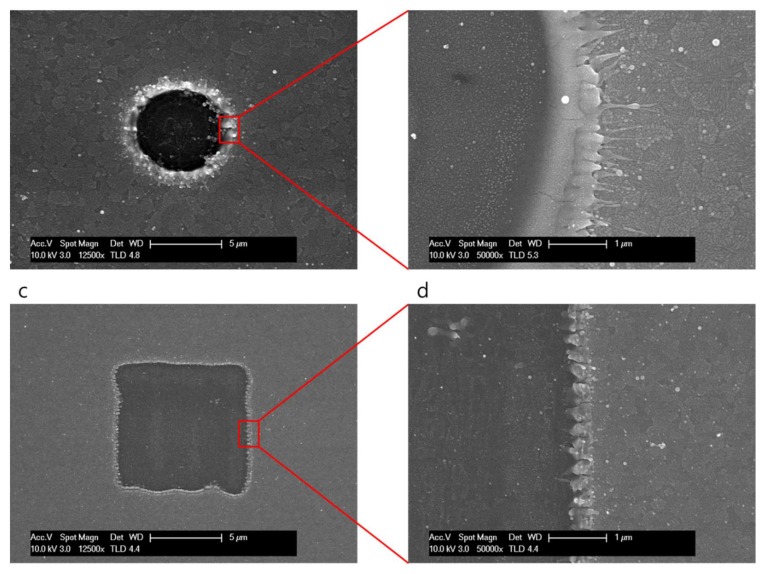
(**a**) Scanning electron microscope (SEM) image of ablation with the Gaussian beam profile; (**b**) Enlarged SEM image of the ablated edge with Gaussian beam irradiation; (**c**) SEM image of ablation with the quasi-flat top beam profile; and (**d**) Enlarged SEM image of the ablated edge with quasi-flat top beam irradiation.

The SEM images of the selectively ablated areas are shown in Figure 2. The morphologies of the ablated ITO thin films were fabricated with the Gaussian beam (Figure 2a), and quasi-flat top beam (Figure 2c) profiles by the irradiation of six pulses. The fluence of the irradiation laser was fixed at 0.9 J/cm^2^, which is higher than the ablation threshold of the ITO thin film (0.24 J/cm^2^), but less than that of the glass substrate (2.1 J/cm^2^); thus, the ITO film was fully ablated and removed from the glass substrate when six laser pulses were irradiated on the ITO thin film, as seen in Figure 2. The widths of the ablated areas irradiated with both the Gaussian beam and squared quasi-flat top beam profiles were approximately 8 μm and 10 μm, respectively; this is important because a 10 μm size corresponds to the size required in patterning OLEDs and solar cells. This square pattern with a size of 10 μm was fabricated by controlling the size of the slit. Figure 2b,d are the zoomed-in SEM images, showing the edge of Figure 2a with the Gaussian beam profile and Figure 2c with the squared quasi-flat top beam, respectively, for more accurate observation. As seen in Figure 2b,d, there is a considerable difference in the surface area of the ablated ridge; in addition, substantial differences in debris and burr were observed between these images. 

**Figure 3:**
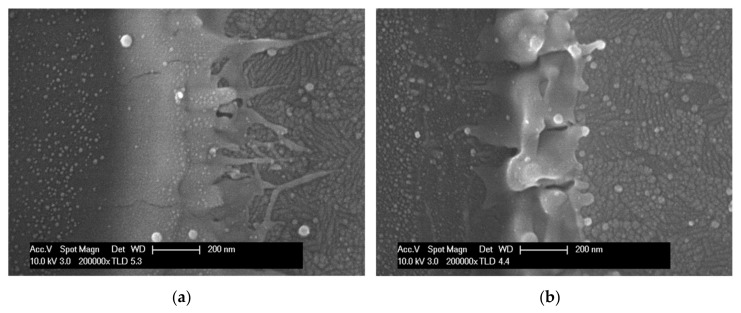
SEM images; Parts (**a**) and (**b**) are the 5× enlarged versions of Figure 2b,d, respectively.

Figure 3 shows the further enlarged SEM images of the Figure 2b,d to closely observe the morphologies of the ablated edge; from these images, it can be observed that the ridge width patterned by the circular Gaussian beam was 1.5–2 times wider than ridge width that was patterned by the squared quasi-flat top beam. In addition, larger quantities of debris were found in the SEM image ablated by the circular Gaussian beam (Figure 3a) as compared with the case in which the squared quasit-flat top beam was used for patterning (Figure 3b). Furthermore, it was observed that the ablation wall in Figure 3a sloped gradually when compared with the wall in Figure 3b. In the two-dimensional (2D) top view (Figure 3a), when patterning with the circular Gaussian beam is observed, crown-shaped spikes are found to the right of the patterned edge, but these were not observed in the case of patterning with the quasi-flat top beam (Figure 3b). These spikes in patterning negatively affect the quality of the packaging. 

**Figure 4:**
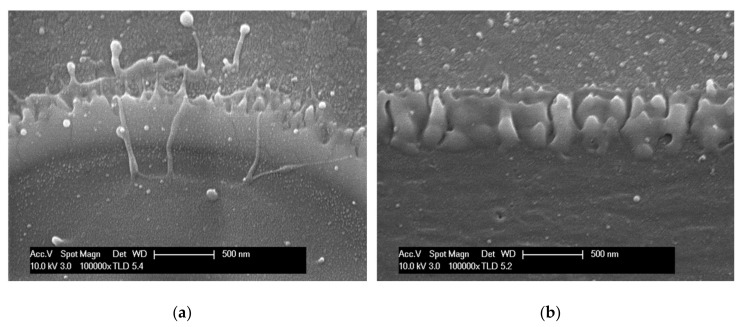
SEM images (**a**) with Gaussian beam irradiation and (**b**) with the quasi-flat top beam, measured by tilting the pattern.

Figure 4a,b are the SEM images that were obtained by tilting the pattern to observe the corresponding cross-sectional views of the top views shown in Figure 3. Figure 4a shows a crown-shaped spike rising sharply, whereas Figure 4b shows a relatively smooth ridge. Furthermore, burr is also observed when patterning with the circular Gaussian beam (Figure 4a, but is absent in the case of ablation with the squared quasi-flat top beam (Figure 4b).

**Figure 5:**
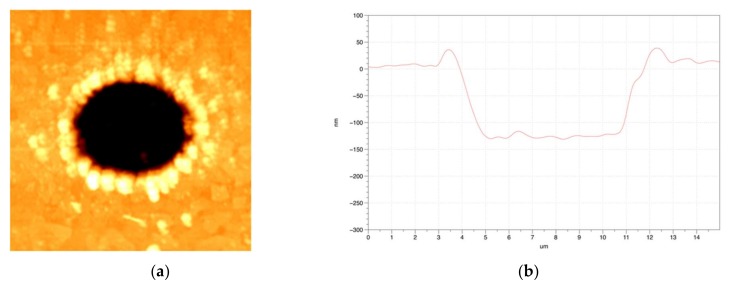

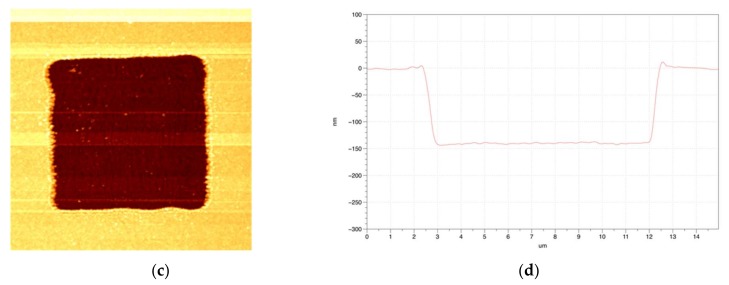
Images of the atomic force microscope (AFM) two-dimensional (2D) morphology data (**a**) with the Gaussian beam profile, and (**c**) with the quasi-flat top beam profile. The cross-section of the ablated area obtained by AFM after femtosecond laser irradiation (**b**) with the Gaussian beam (**d**) with the quasi-flat top beam.

Therefore, the selective ablation of the ITO layer through the glass substrate was successfully achieved by irradiation of laser pulses. The 2D morphologies of the cross-sectional profiles with the use of the Gaussian beam and squared quasi-flat top beam are shown in Figure 5; these morphologies of the ablated area were measured using an AFM. It is clear that the 2D morphologies in Figure 5a,c are the same as those in Figure 2a,c, respectively. Furthermore, the measured 2D depth morphologies of the cross-section of the ablated area are shown in Figure 5b,d. 

**Figure 6:**
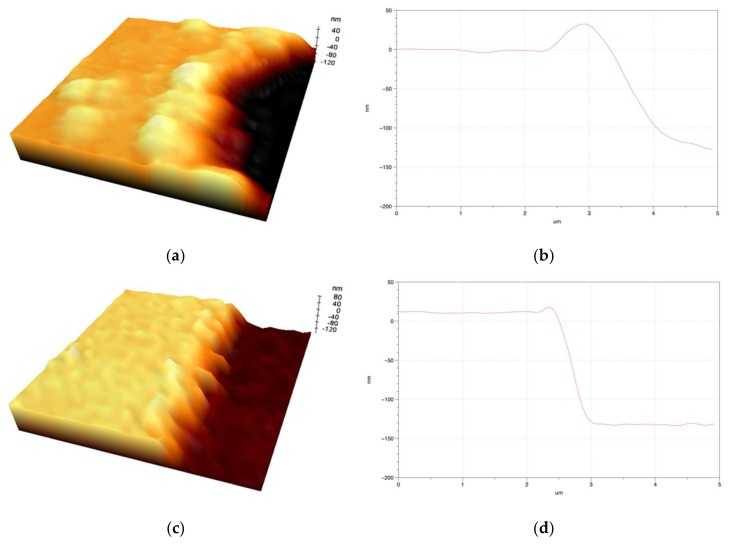
Images of the enlarged AFM three-dimensional (3D) morphology (**a**) with Gaussian beam irradiation, and (**c**) with quasi-flat top beam irradiation. The cross-sectional graph of the ablation edge obtained from the enlarged AFM image (**b**) with Gaussian beam irradiation, and (**d**) with quasi-flat top beam irradiation.

Figure 6 shows the AFM image obtained by further enlarging the edge in Figure 5 to obtain a more quantitatively accurate result. Figure 6a,c show the three-dimensional morphologies of the cross-sectional profiles of the ablated edge by the Gaussian and quasi-flat top beams. The edge morphologies and the ridge areas were obtained with the AFM; a thick broad ridge of edges was observed. Furthermore, Figure 6b,d show the measured 2D graph of the cross-sectional profiles in the ablated edge. The measured ridge height and the width of the circular ablation after irradiation with the Gaussian beam profile were approximately 39 nm and 1.1 μm, respectively; in contrast, for the quasi-flat top beam profile, these were approximately 7 nm and 0.25 μm, respectively. Thus, our quantitative analyses show that a reduction of the ridge width by about five times, and its height by about four times with quasi flat-top beam irradiation is achieved. Moreover, it was observed that the width of pattern walls that were ablated with Gaussian beam is approximately 1.55 μm, whereas it is approximately 330 nm with the quasi flat-top beam, which is considerably smaller. Furthermore, when we ablated a pattern with a width that was similar to that used in optoelectronic devices, the AFM images showed a clear difference in the cross-sectional samples. First, a wider ridge width and higher ridge height in the ablated edge was observed when the Gaussian beam profile was used compared with the quasi flat-top beam; a smaller width and height corresponds to improved quality. Second, a relatively finer edge was observed in the ablated pattern with the quasi-flat top beam irradiation when compared with that obtained with the Gaussian beam irradiation. It was considered that less energy than the ablation threshold was absorbed at the edge of the beam in case of patterning using the Gaussian beam, and heat was generated because of the different energy distribution of the beam. The heat caused melting, and the surface was pushed sideways by this melting. Therefore, as the pulses overlapped, wider and higher ridges were formed. However, narrower and lower ridges were formed when compared with the patterning using the Gaussian beam, due to the uniform energy distribution when using the quasi-flat top beam.

**Figure 7:**
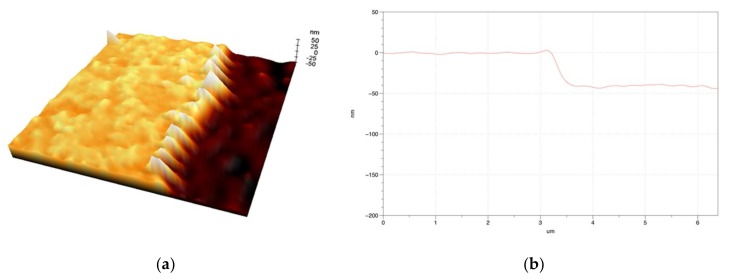
Three-dimensional morphology image (**a**) and cross-sectional graph obtained at the ablation edge using the AFM after one pulse irradiation (**b**).

In addition, a pattern with the ablation depth of around 35 nm was achieved using only one pulse and it was confirmed that ridge height of approximately 3 nm was obtained, as shown in Figure 7. With the transparent electrode thickness of panels used in OLED or solar cell manufacture becoming thinner with time, it is believed that these results will be helpful in the industry.

Several research groups have reported investigations on ITO thin films and ablation structures and these have been applied in various optical waveguides, solar cells, and electrical devices. However, to the best of our knowledge, no previous results have demonstrated well-defined edges and small ridge range on the ITO thin films. In summary, our proposed method using the quasi flat-top square beam profile ablation overcomes the limitations of the conventional method using the Gaussian beam profile considerably, and achieves a ridge length and height that is suitable for OLEDs and solar cells, without the associated disadvantages in ridge morphologies and characteristics, as in the former case.

## 4. Conclusions

We compared the ablation edge morphologies that were obtained by patterning with a femtosecond laser having a wavelength and pulse duration of 1030 nm and 190 fs, respectively, with circular Gaussian and squared quasi flat-top beam profiles. A single-pulse-controlled femtosecond laser beam was used at a fluence of 0.9 J/cm^2^, and the selective ablation of an ITO thin film was achieved after irradiation by six pulses. The squared quasi flat-top beam was produced from a Gaussian beam that was passed through a slit. Based on our experiments, on using the Gaussian beam, the ridge width and height at the pattern edge were approximately 1.1 μm and 39 nm, respectively. In contrast, on using the quasi flat-top beam, the ridge width and height at the pattern edge were approximately 0.25 μm and 7 nm, respectively. This indicates that using the quasi flat-top beam reduced the ridge height and width by four and five times, respectively, when compared with the circular Gaussian beam. Furthermore, it was observed that the width of the ablation wall was approximately 1.55 μm when the circular Gaussian beam was irradiated, as compared with the 330 nm width when the quasi flat-top beam was irradiated, which is about five times narrower. The results of this study can be effectively applied to considerably improve the patterning in the manufacture of various optoelectronic devices.
